# Synergistic Plant Biostimulatory Effects of an Inter-Kingdom Interaction: *Chlorella* sp. and *Kocuria rhizophila* Algal–Bacterial Co-Culture for Sustainable Crop Production

**DOI:** 10.3390/plants15020292

**Published:** 2026-01-18

**Authors:** Katalin Tajti, Attila Farkas, Milán Farkas, Tibor Bíró, Vince Ördög, Gergely Maróti

**Affiliations:** 1 Institute of Plant Biology, HUN-REN Biological Research Center, H-6726 Szeged, Hungary; tajti.katalin@brc.hu (K.T.); farkas.attila@brc.hu (A.F.); 2 Faculty of Science and Informatics, University of Szeged, H-6720 Szeged, Hungary; 3 SeqOmics Biotechnology Ltd., H-6782 Mórahalom, Hungary; 4 Department of Molecular Ecology, Institute of Aquaculture and Environmental Safety, Hungarian University of Agriculture and Life Sciences, H-2100 Gödöllő, Hungary; farkas.milan@uni-mate.hu; 5 Department of Aquatic Environmental Sciences, Faculty of Water Sciences, Ludovika University of Public Service, H-6500 Baja, Hungary; biro.tibor@uni-nke.hu; 6 Department of Plant Sciences, Faculty of Agricultural and Food Sciences, Széchenyi István University, H-9200 Mosonmagyaróvár, Hungary; ordog.vince@sze.hu; 7 Research Centre for Plant Growth and Development, University of KwaZulu-Natal, Pietermaritzburg 3209, South Africa

**Keywords:** plant biostimulation, green alga *Chlorella*, *Kocuria rhizophila*, plant growth promoting bacteria (PGPB), photosynthetic efficiency, extracellular polymeric substances (EPS)

## Abstract

Plant biostimulatory effects of the green alga *Chlorella* sp. MACC-360, the *Kocuria rhizophila* FSP120 bacterial strain, and the combined inter-kingdom co-culture of the alga and bacterium were investigated using *Solanum lycopersicum* as a model plant grown under controlled greenhouse conditions. The application of algal–bacterial co-cultures using the soil drench method significantly improved plant growth parameters, vegetative biomass yield, fruit yield, and photosynthetic performance of the tomato plants. The combined treatment resulted in a 43.7% increase in mean fruit yield, while individual applications of *K. rhizophila* FSP120 and *Chlorella* sp. MACC-360 enhanced yields by 30.85% and 19.44%, respectively. Although total yield increases did not reach statistical significance due to high intra-group variability, the treatment’s efficacy was statistically confirmed through key yield parameters including significantly higher fruit weight and fruit diameter (*p* < 0.05). The enhanced specific biostimulatory effects of the combined treatment could be at least partly attributed to the increased level of algal extracellular polymeric substances (EPS), which was a specific effect of algal co-cultivation with a *Kocuria rhizophila* bacterium. Detailed analysis of plant phenotypic alterations, biomass yield, fruit and flowering parameters, as well as microbial community analysis of the rhizosphere, were conducted and compared among the various treatments. Our results indicate that an appropriately chosen combination and application of biostimulatory microbes can significantly enhance crop production, which might contribute to more sustainable agriculture.

## 1. Introduction

As the world’s population expands, global food supply systems face increasing pressure, while the spread of urban areas continues to limit the amount of land that can be used for agriculture [1]. A solution to these challenges has historically been the utilization of chemical fertilizers, which cause significant environmental harm through the excessive accumulation of nitrogen and phosphorus [2,3]. These accumulated nutrients often lead to ecosystem imbalance, reduced biodiversity, and eventually diminish the economic viability for farmers, as high application rates are increasingly required to sustain yields [4,5]. Given that global food demand is projected to increase by 70–100% by 2050 [6,7], finding novel agricultural methods that sustainably utilize resources—including the reclamation of marginal lands—is imperative [8]. Biostimulants derived from sources like seaweeds, plants, and microorganisms have been identified as key alternatives that enhance crop growth and yield under both normal and stressful conditions [9,10,11]. However, the large-scale preparation of biostimulatory seaweeds and plants is often resource-intensive, risking environmental depletion (seaweed harvesting) or competing for valuable land [12]. Microorganisms offer a compelling advantage, as they multiply rapidly; however, their large-scale production still requires optimized cultivation and processing facilities [13].

The rhizosphere, defined as the soil region immediately adjacent to plant roots, along with the substances exuded there, supports the development of diverse microbial communities due to the release of nutrient-rich root exudates, including vitamins [14]. These exudates serve a dual purpose in aiding plant survival: they can both directly mitigate unfavorable soil conditions while also indirectly strengthening the beneficial functions conferred by root-associated microbial partners [15]. Consequently, certain bacteria within the rhizosphere exert favorable effects on plant growth and confer protection against pathogenic microbes [16].

Plant Growth-Promoting Rhizobacteria (PGPRs), as free-living microbes associated with the plant rhizosphere, play a pivotal role in boosting growth and enhancing resilience to environmental stress. Their operational strategies are diverse: some strains modify root morphology, promoting a larger surface area for enhanced nutrient acquisition [17]. Conversely, other PGPRs may colonize the root cortex internally, forming an endophytic relationship that provides distinct advantages to the host plant throughout its life cycle [18]. Farmers frequently incorporate specific PGPRs, namely *Bacillus* and *Pseudomonas* strains, into cultivation systems [19,20]. These genera are highly valued because their application not only provides essential biofertilizer benefits but also demonstrably enhances crop resilience, minimizing the negative impact of water stress on overall yield [17,21]. Root exudates serve as the key communication bridge for this beneficial interaction, as they chemically mediate the efficacy of PGPRs in the rhizosphere. These root secretions consist of a rich mixture of low-molecular-weight compounds, with tomato exudates, for example, notably containing diverse sugars (e.g., glucose, xylose), various organic acids (such as malic, citric, and succinic acid), and several amino acids (including glutamic acid, aspartic acid, leucine, isoleucine, and lysine) [22,23]. By providing essential carbon sources and acting as chemoattractants, these exudates are fundamental in recruiting beneficial microorganisms to the root surface, thereby supporting the biocontrol processes against root pathogens [24,25]. Specifically, treatments involving *Pseudomonas* strains and mycorrhizae have been shown to enhance key quality metrics such as flowering, fruit production, and the content of both sugars and vitamins, thereby allowing plant growers to reduce reliance on chemical inputs [26]. Furthermore, Horváth et al. (2020) reported that microbial symbiosis significantly boosted productivity during drought periods, noting that green yield saw an increase of approximately one-third, and total carotenoid concentration rose by almost a fifth [27]. In parallel, the *Phylazonit* bacterial blend proved effective across variable weather, yielding larger crops even under conditions of water deficit or surplus moisture [28]. The synergy between PGPRs is also clear: combined use of strains like *Pseudomonas*, *Azotobacter*, and *Azospirillum* demonstrably increases both root and shoot biomass, leading to maximum uptake of vital nutrients (N, P, K) when applied together [29].

Microalgae (MA), which comprise both prokaryotic and eukaryotic organisms, offer promising new tools for agriculture to enhance crop performance, nutrient uptake, and resilience to environmental stress. They have the ability to produce plant hormones, polysaccharides, antimicrobial compounds, and other metabolites that improve agricultural productivity if used for plant or soil treatment [30]. A multitude of microalgae strains have been screened using specific bioassays, and several strains have been highlighted as potential biostimulants. These include several cyanobacterial and green microalgal strains from the genera *Chlorella*, *Scenedesmus*, and *Chlamydomonas*. Their extracts and non-living preparations are mainly applied to plant leaves, while living microalgae are used for soil inoculation. Their uses, biological activities, and applications as sustainable alternatives to synthetic chemicals have been extensively reviewed [31,32,33]. In spite of the high demand, the general use of microalgae-based products is limited by the deficiencies of research in large-scale biomass production, extraction techniques, types of application, field experiments, and development in product formulation and conservation [34].

Both microalgae and PGPRs have the ability to promote plant growth by producing polysaccharides and phytohormones, such as auxin and cytokinin. Furthermore, they can prevent plant diseases by stimulating defense systems and secreting antifungal enzymes and antibiotics. In microalga–bacterium co-cultures, a combined plant growth-promoting and -protecting effect can more easily develop than in monocultures due to the different action mechanisms of the single microbes [35]. Symbiotic interactions of microalgae and bacteria enhance both the yield and diversity of secondary metabolites in the co-cultures. Studies should focus on exploring new combinations of microalgae and bacteria [36]. Specific combinations are published to show how microalgae and bacteria work together to boost plant growth, for example, *Chlorella pyrenoidosa* + *Azotobacter beijerinckii* and *Chlorella vulgaris* + *Bacillus subtilis* in wheat [37].

The benefits of *Kocuria rhizophila* bacterium are based on its capacity to perform crucial PGPR functions, notably phosphate solubilization, IAA production, catalase activity, ACC-deaminase activity, and ammonia production [38,39,40]. Isolates belonging to the *Kocuria* genus have previously been shown to possess broad-spectrum antimicrobial capabilities effective against a range of pathogens [41]. As a powerful biocontrol agent, *Kocuria rhizophila’s* antifungal capabilities are rooted in the synthesis of both bioactive molecules and specific volatile organic compounds (VOCs). These VOCs have proven particularly effective against serious pathogens such as *Fusarium oxysporum* and *Phytophthora cinnamomi* [40]. In this study, the fast-growing and plant hormone-producing strain *Chlorella* sp. MACC-360 was selected for co-culturing experiments with a specific *K. rhizophila* isolate [42]. The *K. rhizophila* FSP120 strain was selected based on its robust plant growth-promoting (PGP) profile and proven efficacy in previous trials [43]. Preliminary screenings revealed that FSP120 exhibited a significant osmotic stress tolerance (10% PEG 6000), IAA production, and phosphate solubilization activity. Notably, FSP120 also demonstrated ACC deaminase activity and a high capacity for biofilm formation, which are critical for rhizosphere colonization and stress mitigation. These traits align with its observed field performance, where FSP120 significantly enhanced plant height and leaf number, advanced the onset of flowering by more than one week, and increased marketable fruit yield by 11–16% under both full and deficit irrigation regimes [43].

Plant tolerance to drought increases substantially as soil water retention improves, a direct result of the physical integrity of the soil being fortified. This crucial fortification stems from extracellular polymeric substances (EPSs) secreted by certain bacterial and algal strains, which actively enhance both soil structure and aggregation, ensuring plants have optimal access to water [44,45]. The presence of EPSs in soil improved nutrient availability by increasing the soil ionic content [46]. Exopolysaccharides also contain sugars that plants can directly absorb and utilize for growth. Additionally, EPSs help microorganisms survive drought conditions by enhancing water retention and regulating the diffusion of organic carbon sources. Furthermore, EPSs can stimulate the secretion of root exudates and aid bacterial adherence to the root surface [45,47]. EPSs are fundamental to rhizobium–legume symbiosis and nitrogen fixation [48]. Furthermore, the presence of algal EPSs may actively facilitate the recruitment of beneficial bacterial and fungal communities within the plant’s rhizosphere and is therefore a critical requirement for plant health [49].

Despite the substantial potential of plant growth-promoting bacteria (PGPB), most existing studies are limited to short-term effects on seedlings in highly controlled laboratory settings. Relatively few investigations track microbial effects over a full crop life cycle under conditions relevant to commercial production. Therefore, the objective of the current study was to address this gap by investigating the distinct and combined biostimulant effects of a eukaryotic microalga and a rhizobacterium on *Solanum lycopersicum*, specifically quantifying their influence on the plant’s photosynthetic efficiency and yield over the entire growth period of tomato crops in a greenhouse environment.

## 2. Results

### 2.1. Basic Algal Growth Curve Studies and Co-Cultivation with FSP120

**Figure 1:**
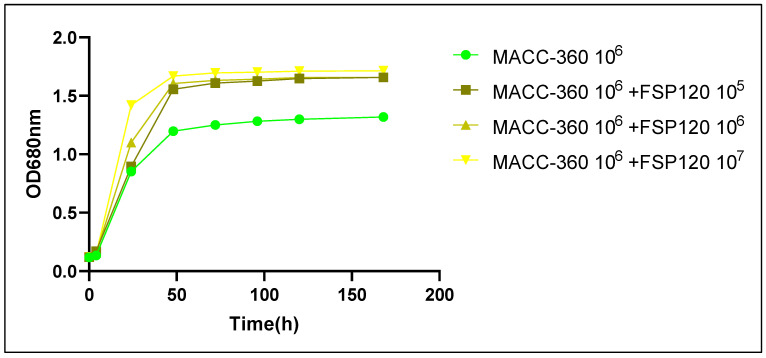
Growth curves of *Chlorella* sp. MACC-360 in axenic culture and in co-cultivation with *Kocuria rhizophila* FSP120 over a 7-day period under light/dark cycle conditions. Algal growth was specifically monitored by measuring optical density at 680 nm (OD_680_), since chlorophyll-a has an absorption peak of 680 nm. Co-cultivation with *K. rhizophila* FSP120 consistently resulted in higher OD_680_ values compared to axenic control, indicating enhanced algal growth.

Co-cultivation of MACC-360 with the bacterium FSP120 enhanced algal growth compared to the axenic culture (Figure 1). Algae-specific OD_680_ measurements indicated that MACC-360 in all cultures shifted to the stationary phase after approximately 50 h; however, they had significantly different maximum OD_680_ values. The axenic alga had an OD_680_ peak of ~1.32, while MACC-360 in co-cultures with FSP120 achieved higher OD_680_ values (~1.73). The stimulatory effect of FSP120 was evident from 24 h and persisted throughout the experiment, suggesting that bacterial presence promoted algal proliferation.

### 2.2. CLSM Analysis of Cell Morphology and EPS Distribution/Co-Culture-Specific EPS Patterns

**Figure 2:**
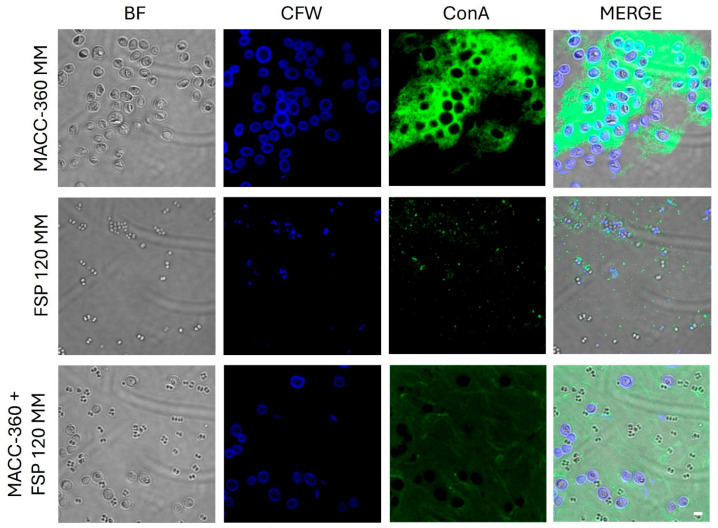
CLSM analyses of an axenic algal strain (*Chlorella* sp. MACC-360), pure bacterial culture (*Kocuria rhizophila* FSP120), and algal–bacterial co-cultures. All samples were stained with CFW (blue fluorescence specific to β-D sugar residues) and ConA (green fluorescence specific to α-D sugar residues). Scale bar in bottom right corner represents 10 μm and applies to all pictures.

The distinct staining characteristics of the algal and bacterial strains, as well as their co-culture, were visualized using CLSM (Figure 2). In the axenic algal culture (*Chlorella* sp. MACC-360), Calcofluor White (CFW) staining revealed a distinct, strong blue fluorescence localized to the cell periphery, corresponding to β-D-glucan components (glucose, mannose) of the algal cell wall. Furthermore, Concanavalin A (ConA) staining exhibited an intense, aggregated green fluorescence closely surrounding the algal cell clusters, indicating the presence of a thick, compact extracellular polymeric substance (EPS) matrix rich in α-D-sugar residues. The pure bacterial culture (*K. rhizophila* FSP120) exhibited distinct blue fluorescence with CFW, indicating the presence of β-D-sugar residues in the cellular structure. In parallel, ConA staining revealed green fluorescent signals that were notably concentrated around bacterial aggregates, while scattered punctate signals were also observed to be freely distributed in the background, albeit less abundantly than in the algal strain. In the algal–bacterial co-cultures, the algal cell walls retained their characteristic blue fluorescence (CFW). However, the structure of the α-D-sugar-rich EPS matrix changed significantly. Unlike the dense clumps observed in the axenic culture, the ConA signal in the co-culture was less intense but more diffusely distributed, covering the entire field of view as a thin, continuous layer.

### 2.3. Surface Morphology and Cell-To-Cell Interactions Analyzed by SEM

**Figure 3:**
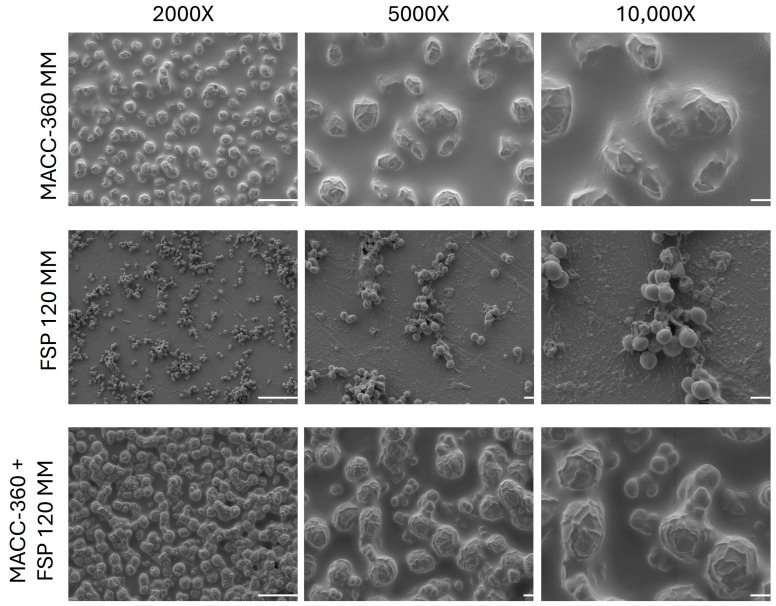
SEM analysis of an axenic algal strain (*Chlorella* sp. MACC-360), pure bacterial culture (*Kocuria rhizophila* FSP120), and algal–bacterial co-cultures. 2000× magnification, scale bars: 10 μm; 5000× magnification, scale bars: 1 μm; 10,000× magnification, scale bars: 1 μm.

To complement the CLSM observations and visualize the ultrastructural details of the interaction, SEM analysis was performed (Figure 3). Axenic *Chlorella* sp. MACC-360 cells appeared as spherical to slightly ovoid structures. Their surface exhibited a rough, textured appearance, and the cells were frequently observed in aggregates, connected by visible extracellular material, which corresponds to the thick, compact EPS matrix identified by ConA staining. The pure *K. rhizophila* FSP120 culture displayed characteristic coccoid morphology. The cells were smaller than the algae and typically arranged in tetrads or irregular clusters. Consistent with the CLSM findings, signs of weak EPS production were observed; unlike the heavy coating seen in the algal culture, only a sparse amount of extracellular material was visible on the cell surfaces and surrounding the bacterial aggregates. In the algal–bacterial co-cultures, a direct physical attachment was evident. Unlike the separate aggregates seen in the pure cultures, the bacterial cells were observed adhering firmly to the surface of the algal cells. This colonization resulted in the formation of mixed aggregates, confirming that the diffuse EPS layer observed in CLSM facilitates a strong structural adhesion between bacterial and algal partners.

### 2.4. Assessment of Algal–Bacterial Biostimulants on Canopy Development and Root Morphology in Tomatoes

**Figure 4:**
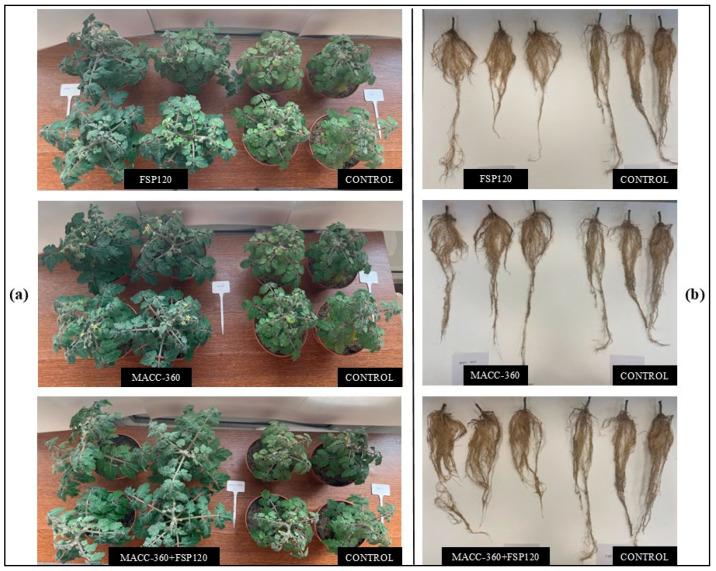
Visualization of the canopy cover, quantified by the area covered by green plant material, was achieved using aerial images taken on the 40th day of growth (Panel (**a**)). Conversely, the root architecture was assessed via frontal views of the roots of 90-day-old uprooted plants, following the removal of the shoot biomass (Panel (**b**)).

Determining plant biomass involved a two-step approach: preliminary assessment relied on the visual evaluation of canopy density, where densely packed plant tissue indicated high biomass and sparse foliage suggested lower biomass. The definitive quantification for all statistical comparisons utilized actual biomass values, which were obtained via destructive harvesting and subsequent dry weight measurements of both roots and shoots. Documentation of these differences included aerial photographs taken on Day 40 (illustrating canopy cover, Figure 4a) and images of 90-day-old uprooted plants (demonstrating root structure, Figure 4b). Crucially, all microbial treatments (FSP120, MACC 360, and the MACC 360 + FSP120 combination) visibly enhanced vegetative vigor and canopy coverage when compared to the MM growth medium control (Figure 4).

Plants treated with FSP120 alone (Figure 4) displayed a moderately larger canopy area and more branched roots than the MM control. However, the MACC-360 and MACC-360 + FSP120 treatments further increased shoot density and branching, resulting in the most profuse foliage and extensive axillary branching. Correspondingly, these treatments also showed a marked increase in root biomass, with the combination treatment producing the most branched root systems.

Overall, plants treated with the MACC-360 + FSP120 combination exhibited the greatest vegetative growth. In contrast, while the bacterial strain FSP120 alone improved growth relative to the MM control, canopy size and root development remained noticeably lower than in either the algal (MACC-360) or the combined treatment, indicating a synergistic effect when bacteria and microalgae were co-applied.

### 2.5. Effect of Microalgal and Bacterial Applications on Plant Morphology

**Figure 5:**
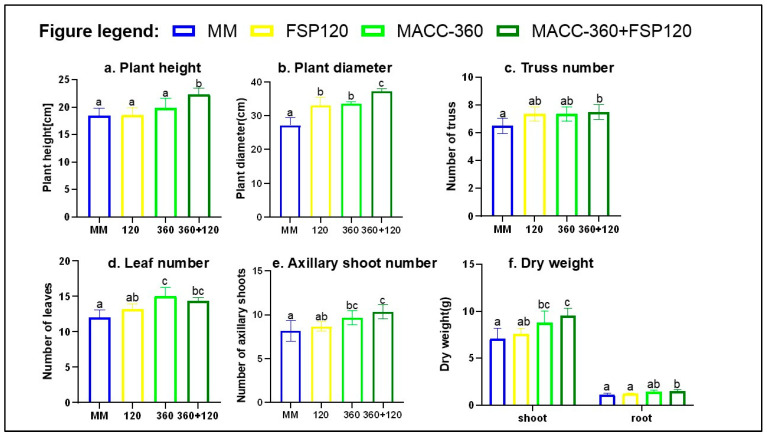
Effects of microalgae and bacterial applications on plants (60-day-old plants); (**a**) plant height; (**b**) plant diameter; (**c**) truss number; (**d**) leaf number; (**e**) axillary shoot number; (**f**) dry weight. Data represent means and standard errors (error bars) of 6 biological replicates per experiment. Different letters on bars indicate significant differences between groups (*p* < 0.05), according to Tukey’s test. One-way ANOVA was used for all parameters.

In addition to leaf dimensions, we evaluated the full spectrum of phenotypic datasets shown in Figure 5 to distinguish physiological variation among the treatments. Specifically, we measured plant height, plant diameter (canopy spread), dry biomass, truss, leaf, and axillary shoot number.

All kinds of microbial inoculation enhanced plant height, plant diameter, truss number, leaf number, and axillary shoot formation compared to the MM control.

The combined MACC-360 + FSP120 significantly enhanced plant height, plant diameter, truss number, leaf number, and axillary shoot formation compared to the MM control, indicating a synergistic effect on overall plant growth. The tallest plants were observed under the combined MACC-360 + FSP120. This was closely followed by MACC-360 plants, while FSP120 slightly lowered this parameter (Figure 5a). All microbial treatments significantly increased plant diameter relative to the MM control (Figure 5b).

The combined treatment *(Chlorella* sp. MACC-360 + *Kocuria rhizophila* FSP120) significantly increased the truss number, whereas MACC-360 and FSP120 alone produced similar numbers (Figure 5c), both above MM. *Chlorella* sp. MACC-360 and the MACC-360 + FSP120 combination had a significant impact on leaf number (Figure 5d). *Chlorella* sp. MACC-360-treated plants had the highest number of leaves, and the combined MACC-360 + FSP120 developed substantially more axillary shoots compared to the MM control (Figure 5e).

Individual application of *Chlorella* sp. MACC-360 significantly promoted shoot dry biomass; MACC-360 alone increased by approximately 24%, while the combined MACC-360 + FSP120 treatment resulted in the highest gain, causing an increase of approximately 33.9% compared to the MM control. *K. rhizophila* FSP120 increased by approximately 7% (Figure 5f).

All treatments including the microalga significantly affected root dry biomass compared to the MM control. FSP120 treatment did not affect root dry biomass significantly, whereas *Chlorella* sp. MACC-360 led to a more pronounced enhancement of 17%. The MACC-360 + FSP120 combination, however, produced the strongest response, with root dry biomass increasing significantly by 27% relative to the MM control (Figure 5f).

### 2.6. Effect of Microbial Treatments on Yield Parameters

**Figure 6:**
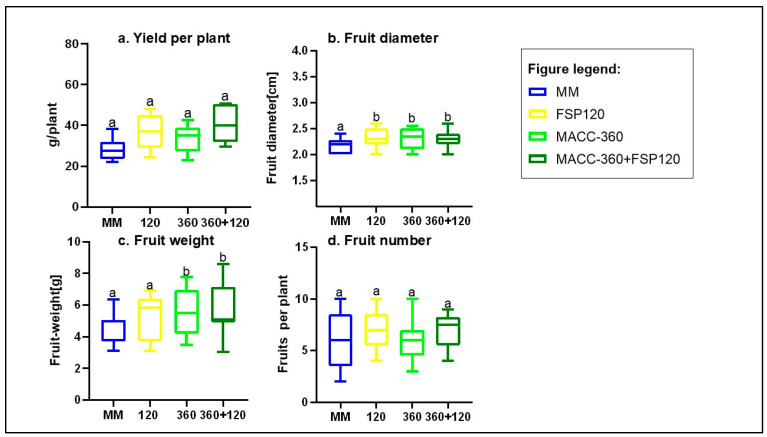
Comparison of the effects of microbial treatment on fruit parameters of plants’ (**a**) yields, (**b**) fruit diameter, (**c**) fruit weight, and (**d**) fruit number. Different letters on bars indicate significant differences between groups (*p* < 0.05), according to Tukey’s test. One-way ANOVA was used for all parameters. Treatments were MM/Control, *Kocuria rhizophila* FSP120, *Chlorella* sp. MACC-360, and the combined treatment MACC-360 + FSP120.

Application of *Kocuria rhizophila* FSP120, *Chlorella* sp. MACC-360, and their combination increased yields, although not significantly. The combined treatment (MACC-360 + FSP120) delivered the highest yield, outperforming controls by 43.7%. The two individual microbial treatments, FSP120 and MACC-360, also demonstrated substantial positive effects on tomato yield when applied alone. FSP120 increased the yield by 30.85%, and MACC-360 resulted in a 19.44% yield enhancement, both figures being considered high and confirming these treatments’ potential as biostimulants (Figure 6a).

*Chlorella*-treated plants had significantly higher fruit weights and fruit diameters, but *K. rhizophila* significantly increased fruit diameter too (Figure 6b,c). None of the microbial treatments significantly affected the fruit number (Figure 6d).

### 2.7. Flowering

**Figure 7:**
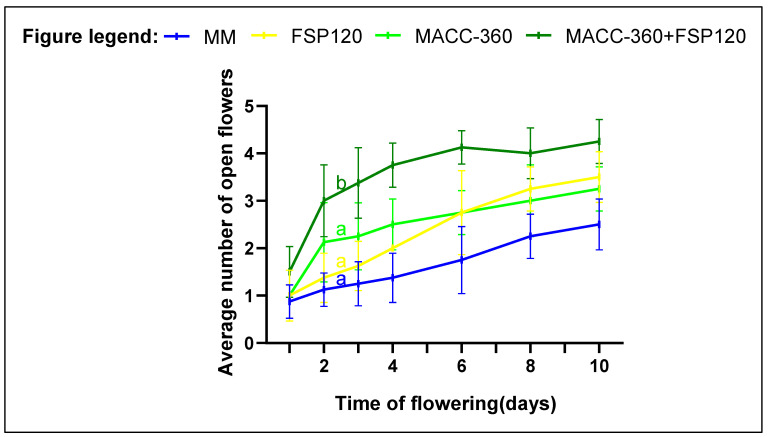
Comparison of the effects of living cells with their growth media as a soil drench on flowering dynamics of tomato plants during the blooming season. The charts show the means of open flowers per plant; different letters show significant differences among treatments based on one-way ANOVA test. Error bars show the standard error of means (SEM). The treatments are *Chlorella* sp. MACC-360, *Kocuria rhizophila* FSP120, a combination of MACC-360 and FSP120, and MM/Control (medium used for cultivation).

In the first week of flowering, co-application of *Chlorella* sp. MACC-360 and *Kocuria rhizophila* FSP120 had the most prominent effect; mix-treated plants maintained a significantly larger number of open flowers relative to the control (*p* < 0.05). The individual application of either MACC-360 or FSP120 enhanced flowering; however, these effects were not significant (Figure 7). The flowering pattern observed may also positively influence fruit development and yields, and it appears that *Chlorella* sp. MACC-360 and *Kocuria rhizophila* FSP120 hardly affected this parameter.

### 2.8. Effect of Chlorella sp. MACC-360 Microalga and Kocuria Rhizophila FSP120 on Photosynthesis

**Figure 8:**
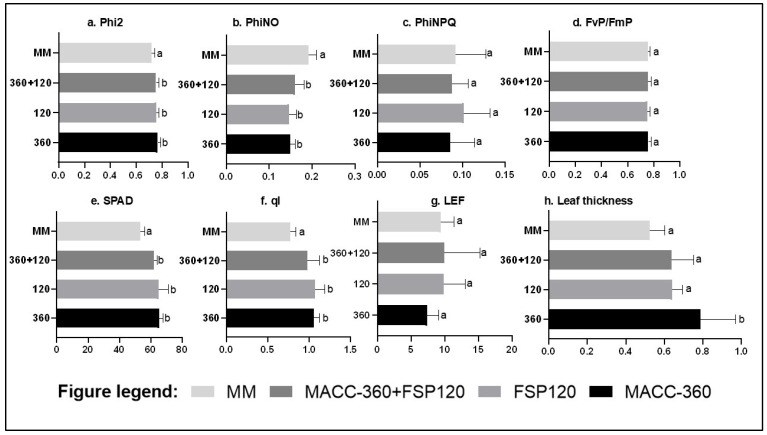
Parameters related to photosynthesis; (**a**) Phi2-PS II quantum yield/ratio of incoming light (excited electrons) used in photochemistry/photosynthesis; (**b**) PhiNO ratio of incoming light (excited electrons) that is lost in non-regulated processes, the products of which can be harmful/cause photodamage; (**c**) PhiNPQ ratio of incoming light (excited electrons) lost through regulated non-photochemical quenching; (**d**) Fv/Fm-maximum quantum yield; (**e**) soil–plant analysis development (SPAD) value, an indicator of plant nitrogen status and relative chlorophyll; (**f**) ql is the fraction of open PS II reaction centers; (**g**) LEF is the linear electron flow; and (**h**) leaf thickness is the thickness of the leaf section clamped by the Multispeq device. Bars show the means; error bars show the SE (standard error) of measurements taken for the first five weeks of growth from 6 plants from each treatment regime. Different letters at the end of bars show significant differences between groups at an alpha of *p* = 0.05 based on ANOVA and Tukey’s test. The treatment regimens are mixed (MM) medium = control medium, *Kocuria rhizophila* FSP120 culture in MM medium, *Chlorella* sp. MACC-360 culture in MM medium, and combined treatment—*Chlorella* sp. MACC-360 and *Kocuria rhizophila* FSP120 culture.

Both the individual strains (Chlorella sp. MACC-360 and Kocuria rhizophila FSP120) and the combination treatment (FSP120 + MACC-360) increased Phi2 (light energy directed to photosynthesis/quantum yield of photosystem II (PS II) and soil–plant analysis development (SPAD)), ql (open PSII reaction centers which indicate the fraction of quinone A (Q_A_) in oxidized state) and leaf thickness, but reduced PhiNO relative to the MM control. Kocuria rhizophila FSP120 increased PhiNPQ and LEF relative to the control. In contrast, *Chlorella* sp. MACC-360 reduced PhiNPQ (regulated non-photochemical quenching) and LEF. Furthermore, the single application of *Chlorella* sp. MACC-360 resulted in the thickest leaves among all treatments (Figure 8).

### 2.9. The Effects of Various Treatments on Microbial Community Composition

**Figure 9:**
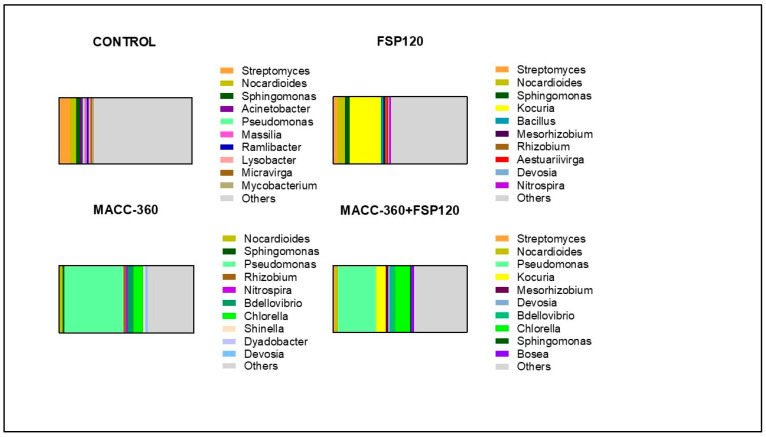
Taxonomic structure of the rhizosphere microbial community at the genus level, including ‘Others’ category.

**Figure 10:**
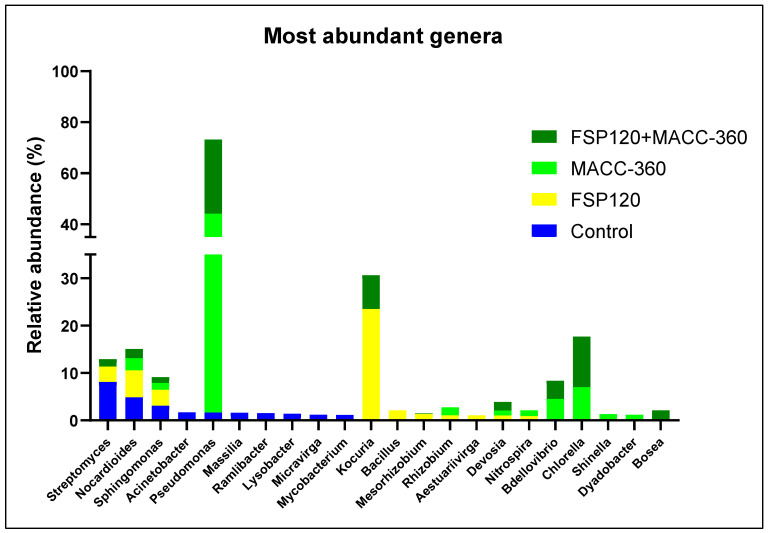
Relative abundance of the dominant genera identified in the plant rhizosphere across different microbial treatments.

Soil metagenomic analysis revealed that microalgal treatments clearly influenced the composition of the tomato rhizosphere microbiome (Figure 9). In soils supplemented with the MACC-360 microalgae (either alone or in combination with *K. rhizophila* FSP120), the relative abundance of a few microbial taxa, including *Pseudomonas* sp., *Bdellovibrio* sp., and *Devosia* sp., significantly increased (Figure 10). The abundance of *Pseudomonas* sp. increased drastically; this genus showed a relative abundance of 44.13% in the *Chlorella* sp. MACC-360 group and was also high (29.06%) in the combined treatment group, representing a dramatic enrichment compared to the control sample (1.67%) and similarly to the FSP120 single bacterial treatment, which resulted in the lowest *Pseudomonas* sp. abundance (0.62%). Interestingly, metagenomic analysis of the control plant’s rhizosphere identified the *Streptomyces* genus as considerably abundant, constituting a 8.12% relative share. In contrast, this abundance was notably reduced across all algal/bacterial treatments: the FSP120-treated plants exhibited a relative *Streptomyces* share of 3.25%, while the combination treatment showed a relative abundance of 1.54%, and the single algal treatment (MACC-360) resulted in the lowest *Streptomyces* abundance at 1.0%. The rhizosphere metagenome analysis also confirmed successful rhizosphere colonization by *K. rhizophila* FSP120. This bacterium showed a relative abundance of 23.5% in the rhizosphere of tomato plants receiving only bacterial treatment, while the abundance of *Kocuria* sp. was 7.14% in the algal–bacterial co-culture-treated samples.

## 3. Discussion

Novel biostimulant strategies are critically required to bolster crop resilience against the intensifying challenges of climate change. This urgency stems from the rise in adverse environmental factors such as elevated temperatures, drought, and extreme weather patterns, which increasingly necessitate innovative solutions in agriculture [50]. In the present study, we observed that tomatoes co-inoculated with a eukaryotic green microalga strain *Chlorella* sp. MACC-360 and a PGPR strain *Kocuria rhizophila* FSP120 showed enhanced plant growth and photosynthetic activity, as well as vegetative biomass and fruit yields, in comparison with non-inoculated plants. Members of the *Kocuria* genus are aerobic, non-encapsulated, Gram-positive, and coccoid bacteria belonging to the order of *Actinomycetales*. *Kocuria* isolates were collected from various sources, such as air, freshwater, plants’ rhizosphere, and seawater and marine sediment [51]. *Kocuria* sp. LSM1-65 showed a siderophore-producing capability [52]. The *K. rhizophila* treatment proved to be a promising approach for improving the salt stress tolerance of wheat [53]. *K. rhizophila* Y1 was shown to effectively produce IAA and to solubilize calcium phosphate [54]. Effects of microalgae on tomato plant growth, yield, and fruit properties were investigated earlier. *Chlorella vulgaris* green algae treatment had a significant positive effect on average tomato fruit weight and fruit diameter [55,56]. Treatments with microalgae extracts significantly increased the production of antioxidant molecules in different types of quinoa and bolstered salt stress tolerance [57].

Both the *Kocuria rhizophila* FSP120 and *Chlorella* sp. MACC-360 treatments had significant positive effects on tomato plant height, leaf number, flowering, and fruit parameters when applied through the soil drench method. However, the strongest positive effects were observed when the green alga and the PGPR bacterium were applied together. This might be explained by the synergistic relationship between the selected alga and bacterium. The co-cultivation of MACC-360 and FSP120 led to enhanced algal growth, as confirmed by elevated specific algal growth in the co-culture compared to the axenic alga culture. This mutualistic effect implied a delicate metabolic relationship between the two microorganisms, which was indicated by the specifically altered pattern of EPS production in the co-cultures. The observed synergy is consistent with previous results demonstrating that bacterial partners supply crucial metabolites, such as essential vitamins and other growth-promoting compounds to significantly boost microalgal proliferation and EPS production [58]. Microbial treatments, particularly the combined algal–bacterial application, significantly enhanced overall vegetative growth and biomass accumulation of the tomato plant. The MACC-360 + FSP120 co-inoculation resulted in the highest shoot biomass and axillary branching. Root biomass also exhibited a notable increase, with the combined treatment yielding the strongest response. However, it is noteworthy that while the root dry weight was higher in the co-culture-treated plants compared to the controls, the roots of these plants were observed to be shorter and had more branches compared to the non-inoculated plants. While direct phytohormone quantification was not performed, these morphological shifts, especially the increased branching, are consistent with the typical effects of microbial auxin production. This points toward a potential mechanism where the microalgal partner may metabolically support the PGP bacterium’s hormone synthesis, a pathway frequently observed in PGPB interactions [42,59].

*Kocuria rhizophila* FSP120 treatment alone had a moderate or slightly suppressive effect on plant height, whereas MACC-360 alga treatment alone provided modest improvements for multiple parameters, reflecting strain-specific impacts on growth promotion [60]. The co-applied plants maintained earlier a significantly higher number of open flowers. The individual application of either MACC-360 or FSP120 enhanced flowering; however, these enhancements were not statistically significant. The combined impact of the two microbes points toward a potential stimulation of phytohormone synthesis, such as auxins or gibberellins, which are known to be crucial for floral initiation and development [61,62].

All microbial treatments (*Chlorella* sp. MACC-360, *Kocuria rhizophila* FSP120, and the combination) uniformly improved overall photosynthetic efficiency. However, the two strains adopted differential strategies for managing light energy. *Chlorella* sp. MACC-360 and the combination treatment maximized the photochemical yield by reducing the need for photoprotection (PhiNPQ and LEF decreased), maintaining a highly oxidized QA pool. In contrast, *Kocuria rhizophila* FSP120 actively favored a photoprotective strategy, increasing energy lost via regulated means (PhiNPQ increased) while still achieving high linear electron flow (LEF increased) and low unregulated loss [63]. These results clearly demonstrate the strain-specific differential effects of the two microbes on photosynthetic performance and photoprotective mechanisms in tomato plants. The microbial applications of both the microalgae and bacteria significantly enhanced the plants’ capacity for nutrient acquisition, a conclusion strongly supported by the higher observed SPAD readings [64].

Based on the metagenomic analysis, changes were identified in the tomato rhizosphere microbial communities in response to the microbial treatments (either single or co-inoculations). The applied *Chlorella* alga and *Kocuria* bacterium strains appeared to alter the overall rhizosphere bacterial community structure. The microbiome compositions showed characteristic shifts in response to the individual and co-application of the alga and bacterium. In our samples, microalgal treatment was associated with an enrichment of the *Pseudomonas* genus in the rhizosphere, which potentially influenced the microbial community structure [64]. This recruitment may be vital because members of this genus (especially *Pseudomonas putida*) are broadly known for their plant growth-promoting (PGP) effects. The *Pseudomonas*-related PGP mechanisms include ACC-deaminase activity, indole-3-acetic acid (IAA) synthesis, and phosphate solubilization. Studies, such as Jin et al. (2022), have confirmed these beneficial impacts by isolating a specific *P. putida* strain from the *Cercidiphyllum japonicum* rhizosphere [65]. This suggests a role for *Pseudomonas* in nutrient mobilization and stress mitigation for plants. Furthermore, the presence of *Pseudomonas* bacteria is known to significantly improve nutrient uptake in other crops as well, e.g., in maize and soybean [66]. A relatively high abundance of the *Streptomyces* genus was observed in the control plant’s rhizosphere. This is well in line with the nutrient limitations inherent in the applied sandy growth environment, which represented sub-optimal conditions for plant growth. However, the presence of *Streptomyces* species and their protective functions were retained in all conditions (although with lower relative abundance in the case of algal and microbial treatments), indicating the importance of their plant growth-promoting (PGP) traits, especially the effective production of phytohormones and siderophores [67].

The observed shifts in the rhizosphere microbiome correlate with the structural changes in the *Chlorella* sp. MACC-360 EPS matrix in the presence of *Kocuria rhizophila* FSP120 bacteria. This inter-kingdom interaction represents a potential mechanism for improving soil–water dynamics in the rhizosphere. Although EPS was not quantified biochemically, the visual evidence of altered biofilm architecture suggests a pathway by which the co-culture may indirectly (through re-structuring the rhizosphere microbial community) support the superior performance (vegetative biomass and fruit yield) recorded in the co-inoculated plants. Such microbial matrices, especially in sandy soils, are known to improve soil hydrological characteristics such as by absorbing moisture and retaining water in topsoil [66], providing a plausible explanation for the observed biological benefits.

The notable 43.7% numerical increase in tomato yield following co-inoculation with MACC-360 and FSP120 suggests a promising biological trend and justifies the use of microbial combinations as biostimulants. Although this increase was not statistically significant due to high individual plant variability, the effect is consistent with other agricultural studies. For instance, Kopta (2018) reported that combined bacterial and freshwater algae applications led to substantial gains in lettuce weight (18.9% and 22.7%) [68]. While our findings point toward the potential of combining microbial strains for yield enhancement, further lab and field trials are required to confirm the statistical robustness.

## 4. Materials and Methods

### 4.1. Strains and Cultivation

The microalga *Chlorella* sp. MACC-360 strain was obtained from Mosonmagyaróvár Algal Culture Collection (MACC; Institute of Plant Biology, University of West-Hungary, Hungary) and maintained on mixed media (MM) plates (TAP and PCA media added in an 85:15 ratio). The *Kocuria rhizophila* FSP120 strain, which is maintained in the collections of the Hungarian University of Agriculture and Life Sciences (MATE), was isolated in 2021 from the root surface of *Festuca rupicola*, the dominant plant species of an arid Hungarian steppe [32].

The bacterial strain *Kocuria rhizophila* FSP120 was maintained on Reasoner’s 2A (R2A) agar plates. Starter cultures (50 mL) were prepared in MM medium and incubated for 7 days, then scaled up into 1 L flasks containing MM medium and grown for an additional 7 days under shaking conditions.

The algal strain *Chlorella* sp. MACC-360 was maintained on TAP agar plates. For liquid culture, 15 mL of MM medium in a 50 mL Erlenmeyer flask was inoculated with cells from a fully grown plate and incubated for 5 days (25 °C, 16:8 h light/dark cycle, 180 rpm). Then, 5 mL of this starter culture was transferred into 50 mL MM medium in a 100 mL flask and grown for 7 days. Finally, 50 mL of the culture was inoculated into 1000 mL MM medium in a 1.5 L flask and incubated for 7 days. The resulting culture suspension of FSP120 and MACC-360 was used for soil treatment in plant pots.

### 4.2. Growth Curve Measurements

Growth curve analyses were conducted to assess the growth dynamics of *Chlorella* sp. MACC-360 in monoculture and in co-cultivation with *Kocuria rhizophila* FSP120. Algal cells were cultured for 5 days in MM medium under continuous shaking at 200 rpm and a light intensity of 50 μmol m^−2^ s^−1^. TAP and PCA media added at an 85:15 ratio composed the mixed media (MM) employed in this study. MM was used to culture both algal and bacterial cells to keep culture conditions the same. Previous studies in the lab and preliminary studies in this project confirmed that most bacteria did not grow in only TAP. This was overcome by a small amendment to PCA media components in TAP media.

The bacterial strain *Kocuria rhizophila* FSP120 was cultured overnight in MM medium at 30 °C. Bacterial stock inocula were prepared by adjusting the cell density of the overnight cultures to 1 × 10^5^, 1 × 10^6^, and 1 × 10^7^ cells mL^−1^.

Growth assays were performed in 48-well microplates using a HIDEX Sense microplate reader. Each well contained 650 µL of MM medium, 150 µL of algal inoculum standardized to 10^6^–10^8^ cells mL^−1^, and 50 µL of bacterial inoculum at the corresponding concentrations. All treatments were conducted in triplicate.

Optical density at 680 nm (OD_680_) was measured at the start of the experiment (0 h), at 4 h, and then daily for 7 days using the HIDEX Sense reader. Average OD_680_ values were plotted and analyzed in GraphPad Prism 8.0 to generate growth curves.

### 4.3. Determination of Cell Numbers

Both the algal and bacterial cells were counted using automated cell counters before plant treatments. The LUNA-FL (Logos Biosystems, Anyang, South Korea) dual fluorescence cell counter was used with the red fluorescence channel. When chlorophyll molecules are excited, they emit red fluorescence. This can be used as a proxy for automated counting of live algal cells. Bacterial cells were enumerated using the QUANTOM Tx (Logos Biosystems, Anyang, Republic of Korea) microbial cell counter using live cell staining.

### 4.4. Morphological Studies

Confocal Laser Scanning Microscopy (CLSM, Olympus FluoView FV-300, Olympus Optical Co., Ltd., Tokyo, Japan) was used in this study. Aliquots of 50 μL of culture were collected into Eppendorf tubes and stained with Calcofluor White (CFW) and/or with Concanavalin A (ConA) at a final fluorescent dye concentration of 10 µg/μL. After a 30 min incubation in the dark, the samples (8 μL) were spotted onto microscope slides, covered with a 2% (*w*/*v*) agar slice, and observed with an Olympus Fluoview FV 1000 confocal laser scanning microscope (Tokyo, Japan) equipped with a 63× magnification objective and 4× optical zoom. Sequential scanning was used to avoid crosstalk between the fluorescent dyes and chlorophyll autofluorescence.

Scanning electron microscopy (SEM) was used to investigate the co-cultures and their EPS production in detail. Cells were fixed with 2.5% (*v*/*v*) glutaraldehyde and 0.05 M cacodylate buffer (pH 7.2) in PBS overnight at 4 °C. Five microliters (5 µL) of the algal and bacterial suspensions were spotted onto a silicon disk coated with 0.01% Poly-L-Lysine. The disks were washed twice with PBS and dehydrated with a graded ethanol series (30%, 50%, 70%, 80%, and 100% ethanol, each for 1 h). Then, the samples were incubated in hexamethyldisilazane, a chemical drying reagent. The chemically dried samples were coated with a 12 nm layer of gold and observed under a JEOL JSM-7100F/LV high-end field emission scanning electron microscope (Tokyo, Japan) at 250×, 1500×, and 10,000× magnification.

### 4.5. Plant Growth Experimental Design and Treatments

*Solanum lycopersicum* L. seeds of the Vilma variety were used for the plant growth studies. All tomato seeds were surface sterilized by 10 min of soaking in 5% sodium hypochlorite solution, followed by five successive rinses with sterile distilled water. The seeds were allowed to imbibe water for about 2 h. The seeds were directly sown into 12 cm diameter pots containing a moist sand–soil mixture in a 2:1 ratio (*v*/*v*). This ratio was specifically chosen to create a nutrient-poor growth medium for the tomato plants.

Germination and subsequent early seedling development were conducted in a controlled-condition greenhouse environment for one week. Throughout the experimental period, the pots were watered two times with Solution 1 diluted 40-fold. Solution 1 was prepared from the stock solution with the following nutrient solutions: KNO_3_, 15 mM; Ca (NO_3_)_2_ 4H_2_O, 12.5 mM; Ca(H_2_PO_4_)_2_, 1 mM; MgSO_4_ 7H_2_O, 1 mM; Fe EDTA, 0.01 mM; MnCl_2_, 0.004 mM; H_3_BO_3_, 0.02 mM; ZnSO_4_ 7H_2_O, 0.0004 mM; NaMoO_4_, 0.0001 mM; and CaSO_4_ 5H_2_O, 0.0001 mM. Plants were grown in the greenhouse at 24–26 °C and with a 16 h photoperiod. Each treatment had a total of 6 plants: 1 plant per pot, 6 pots per treatment. There were 3 pots placed on a tray, and a treatment included 2 trays. To ensure the uniform exposure of all plants to environmental factors, the experimental trays were periodically moved and rotated on the bench. Strict adherence to the established guidelines was followed for all treatments applied to the soil and plants, which were organized according to a randomized block design.

Four experimental groups were established: three containing microbial treatments and one untreated control (MM growth medium). The sand-soil mixture of pot plants in the treatment groups was inoculated with suspensions of the microalgal strain *Chlorella* sp. MACC-360, the bacterial strain *Kocuria rhizophila* FSP120, or a combination of both MACC-360 and FSP120. These simplified notations will be used throughout the remainder of this manuscript. Inoculations were performed weekly starting from 15 days after sowing for a total of three applications (Days 15, 22, and 29). This timing was strategically chosen to cover the critical developmental window between seedling establishment and the onset of flowering. Each plant received 100 mL of whole algal or bacterial or algal–bacterial cultures via soil drenching.

All cultures were standardized to an approximate cell concentration of 10^7^ cells mL^−1^, a density determined as optimal in preliminary trials to ensure a consistent biostimulatory response. The combination treatment had the same total cell number, half of them being bacteria and half of the cells being algae. After 40 days, aerial photographs were taken to document the plant canopy development. Growth experiments were terminated after 90 days, and six plants from each treatment were selected for phenotypic analysis. Flowering was considered to start on Day 0 (D0) when the first open flower appeared. Starting from this point, the daily number of open flowers per plant in each treatment was recorded to analyze the flowering kinetics.

For the determination of dry biomass in the plants, the following protocol was implemented: Plants were carefully uprooted, and the roots were thoroughly rinsed with distilled water, facilitating the elimination of all adhering soil debris. Following separation of the shoots and roots, individual weighing occurred. All plant material was then placed in a dry air oven at 70 °C for 48 h, enabling quantification of the final dry biomass.

### 4.6. Photosynthetic Activity Measurement

Fluorescence-based measurements of photosynthetic parameters were performed on plant leaves on a weekly basis with the Multispeq (PhotosynQ Inc., East Lansing, MI, USA) hand-held device [69]. All measurements were conducted between 9:00 and 11:00 a.m. on the same section of the 5th leaf. The photosynthetic activity of the plants was assessed using several parameters. Fluorescence measurements included the maximum quantum yield (Fv/Fm), representing the potential photochemical efficiency of PS II, and the actual quantum yield (PhiII) of PS II, indicating operational efficiency. Energy partitioning was evaluated using the non-regulated energy loss (PhiNO) and the regulated non-photochemical quenching (PhiNPQ), which quantifies the proportion of excess light energy harmlessly dissipated as heat. The PS II openness was determined by the fraction of oxidized PS II reaction centers (qL). Additionally, the relative leaf chlorophyll content was quantified using the SPAD index (soil–plant analysis development), and leaf thickness was recorded as a key morphological parameter [69,70].

### 4.7. Total DNA Extraction from Samples

The effect of the treatments on the microbial community was assessed by metagenomic sequencing. Rhizosphere samples were collected after the last treatment for 2 weeks. To ensure a representative profile, rhizosphere soil was collected from all six replicates per treatment using a non-destructive sampling technique and then pooled into a single composite sample for analysis. This approach allowed the plants to remain intact for subsequent fruit yield measurements. Rhizosphere soil was sampled directly from the root zone, i.e., soil tightly adhering to the roots. Soil particles larger than 1 mm were carefully removed from the root surface using sterile tweezers, after which approximately 2 g of fine roots were transferred into sterile conical centrifuge tubes. Each tube received 10 mL of 1× phosphate-buffered saline (PBS; 1.44 g Na_2_HPO_4_, 0.2 g KCl, and 0.24 g KH_2_PO_4_ per liter of deionized water, pH 7.4). The microbial cells adhering to the root surface were detached by vortexing twice for 10 s. The resulting suspension was filtered through a sterile 200 μm mesh to remove plant debris, and the filtrate was centrifuged at 4600 rpm for 15 min. DNA isolation from the samples was performed using the Zymo Quick-DNA Fecal/Soil Microbe Miniprep Kit (Zymo Research, Irvine, CA, USA) according to the manufacturer’s protocol. Total soil DNA quality was assessed using an Agilent Tapestation 4150 (Agilent Technologies, Santa Clara, CA, USA) capillary gel electrophoresis system, and DNA concentration was measured by a Qubit fluorometer. For NGS analysis, the recommendations of the Illumina sequencing platform were closely followed (Illumina Inc., USA). DNA samples were used for in vitro fragment library preparation using the NEBNext Ultra II Library Prep Kit (New England Biolabs, Ipswich, MA, USA). Metagenome sequencing was performed on an Illumina NovaSeq platform using the NovaSeq 6000 SP Reagent Kit (Illumina, San Diego, CA, USA) v1.5 and a 2 × 150 nt PE chemistry.

### 4.8. Statistical Analysis

Prior to conducting analyses of variance (ANOVAs), data were assessed for normality and homogeneity. Depending on the experimental design, one-way ANOVA models were applied to evaluate treatment effects. When significant differences were identified, means were compared using Tukey’s Honestly Significant Difference (HSD) post hoc test, with statistical significance set at *p* < 0.05.

## 5. Conclusions

Co-inoculation with *Chlorella* sp. MACC-360 and *Kocuria rhizophila* FSP120 consistently enhanced growth, photosynthetic efficiency, biomass, and fruit yields of tomato plants compared to the control. Although the fruit yield increase was not statistically significant due to high intra-group variability, the combined treatment resulted in significantly higher tomato fruit weight and diameter (*p* < 0.05). Extracellular polymeric substances (EPSs) produced either by the algae or the bacterial partners (which had especially high levels in the co-cultures) are hypothesized to have a major role in the improved plant growth and photosynthetic parameters, most probably through the alteration of the rhizosphere microbial composition. The results reveal the hidden potential of combining various microbes, including bacteria, fungi, and algae, for use as natural fertilizers in crop production. Efficient and rapid screening systems to identify high-performing microbial communities are crucial for the development of novel biostimulatory products.

## Data Availability

The data presented in this study are available on request from the corresponding author. The raw data supporting the conclusions of this article will be made available by the authors on request.
